# Remarks on Fever

**Published:** 1823-05

**Authors:** D. Johnson

**Affiliations:** Great Torrington.


### Art. IV.—*Remarks on Fever*. By D. Johnson, Esq. of Great Torrington.

In order to trace fevers as near as possible to their source, I shall begin with colds, which are often the origin of fevers, and are the most common of all complaints. They are not always produced, as is generally supposed, by exposure to a cold atmosphere, a current of air, or by wetting the feet or other parts of the body: therefore, I conceive the term is not always applicable. Cold often causes a diminution of perspiration, and also discharge of vapour by exporation: the consequence is a fullness in the system, and nature seeks relief from it by a discharge from the nostrils, fauces, lungs, or a looseness from the intestines; and all these discharges are attended with more or less inflammation. A cough is often produced by a discharge from the nose or throat falling on the glottis, and oozing into the trachea and irritating it; which should always be distinguished from such as is caused by viscid secretions in the lungs and trachea.

The same symptoms that attend colds, I conceive, are often produced by fulness of habit, without any influence whatever of cold; therefore the term does not always convey a just idea of the illness. Such complaints, as well as colds, are always attended with febrile symptoms; and, if nature or art does not soon relieve the system of fulness, a regular fever is the consequence; and all fevers become putrid, if not soon relieved by some great effort.

Many fevers are brought on by obstructions in some particular gland or glands, most probably owing to fulness of habit, over-feeding, bad food, or constitutional ill humors. Such obstructions will often continue for a long time without discovering themselves, or the constitution making any effort of consequence for relief; and the person, though not feeling his usual vigour, does not often complain, until the obstruction impedes the regular functions of the body that sustain life,—then nature endeavours to relieve itself by producing a fever. For example —the mesentery or other glands, in or communicating with the intestines, being obstructed, will cause costiveness, which may continue for a long time without producing any serious disease, till at last, for want of a due discharge of the bile secreted, the liver, gall-bladder, and biliary ducts, become gorged, and the quality of the bile is injured from retention. In this case, the liver becomes the ostensible, or rather proximate, cause of fever, though not the primary, and *vice versâ.*

Intermittent fevers, I also think, are owing to obstructions: the causes of them may be the same as with common fevers; but it strikes me that the immediate cause of the fit is owing to a cessation of the digestive process. If I may be allowed the comparison, I would compare it to a machine, the wheels of which require a regular supply of oil to prevent friction, and keep them in constant motion, without which they would soon stop. If any of the glandular secretions, requisite to carry on the digestive process, are obstructed, that process is not only stopped, but the general circulation of the blood is also impeded, which accounts for the cold fit; and the hot fit is an effort of nature to remove such impediment, which it effects, and at the same time causes the secretion to flow, without entirely removing the obstruction; yet sufficient is discharged to carry on digestion for a short time, and, when a fresh supply is required, another fit ensues; the general interval between each fit being a day and night, sometimes more, particularly when the disease is going off. I have always considered sleep necessary to good digestion, as watchfulness impedes it; and indigestion from any other cause will generally occasion watchfulness or incubus: this, I think, accounts for the regular return of agues. Remission in fevers may sometimes be owing to the same cause. There exists a considerable connexion and sympathy between the skin and glands of, and connected with, the intestines; which induces me to think that obstruction in the former is the remote, and in the latter the proximate, cause of agues.

An emetic taken at the commencement of the cold fit, will shorten its duration, and also cause the hot fit to pass off quicker: from this I conclude, that the exertion of vomiting causes the glandular secretions to flow largely into the intestines, and gives energy to the heart and general circulation of the blood, removing the constriction on the small vessels and pores of the skin. I once laboured under a quotidian for nearly a twelvemonth, from which I was entirely relieved by a violent flux. During the time I had the ague, I often experienced the benefit of emetics, as before recited: derangement of the liver appeared to be the cause, for which I took mercurials, and experienced no relief until the flux came on.

All medical men must have observed that in continued fevers the digestive process is never carried on as it should be: indeed, I may say that it is almost entirely suspended, for solid food taken at such times passes off little altered, and in its passage produces irritation and uneasiness; but, as soon as a crisis has taken place, the person craves food, and then digests it almost as well as if he were in perfect health; which, I think, shows the wonderful efforts of nature for her own relief, and strongly points out the impropriety of giving food in fevers, and in a great measure justifies the practice followed by the native doctors in India, of not allowing any thing but water to be taken in fevers until the ninth day.

I consider all fevers are efforts of nature, and almost every symptom attending them are similar efforts, and therefore should be carefully observed, and never obstructed, in their operations. They are at all times our best guides; yet it sometimes happens that these efforts become diseases, or rather what is termed ostensible or proximate causes of diseases, which of themselves are of more serious consequence than their causes. To enter into a particular description of them, or of all the different fevers, is not my intention. It may be observed of diseases, like occurrences in life, that apparent evil is often intended for good.

In some fevers, putrescency commences in the early stages; and such fevers are now commonly distinguished by the appellation of Typhus, which, as I understand it, implies that they are supposed to arise from miasma, and are infectious; but the term is often misapplied. In common fevers, putrescency does not commence at the same period in all subjects; but after a certain time it becomes necessary with all, to relieve the system, when nature and art have failed in all other efforts; and then I am at a loss how to distinguish them from typhus. By putrescency, the blood becomes somewhat dissolved, or its particles are broken and divided, by which all the large glandular secretions are increased, particularly such as empty themselves into the intestinal canal, relieving the vascular system of its fulness; which evidently points out the necessity of keeping the bowels regularly open with purgatives, and, to me, clearly shows why reduction by these means is preferable to bleeding, which takes away the vital and pure blood with the impure; whereas, purgatives carry off only the impure. I would wish to be understood, that I think bleeding is often absolutely necessary when there is considerable fulness, or when urgent symptoms require immediate relief.

I am of opinion that mercury taken into the system acts much in the same manner as natural putrescency, by producing it artificially, and acting particularly on the liver and parts connected with it, and on that part of the blood proper for the secretion of bile, which, in consequence, is secreted of a less viscid quality. To such action I attribute the good it does in most bilious complaints, and in some fevers, together with its causing the bowels to empty themselves regularly: by these means lessening the quantity, and attenuating the quality, of the general mass of blood; removing the constriction on the pores of the skin, and thereby enabling them to resume their natural functions.

For the foregoing reasons, I am confident that, to use any violent means to check putrescency, is often wrong; therefore I am not an advocate for cold ablutions. In fevers, I have never witnessed the re-action which takes place in vigorous constitutions in health after cold-bathing, although so much is said about it; and, if it does not take place, causing a general glow or moisture on the skin, in my opinion it must add to the constriction already on it; and, although it may abstract heat, it will obstruct the process of nature for relief, and by that increase the disease. Tepid ablutions, according to my judgment, would be following the dictates of nature, and more likely to be beneficial. I am aware that strong objections may be made to this practice, and also to the free use of mercury, from the idea of its debilitating and tending to increase the putrescency already much dreaded, and which, if carried beyond a certain point, will cause dissolution. This, I admit, is a point of important consideration for the medical practitioner; and I cannot help thinking that many errors are committed from the dread of putrefaction, without taking into consideration that a certain state of it is often necessary to produce a favourable crisis. Most medical practitioners have witnessed cases in which their patients have appeared to be in the last agonies, with very little hopes of recovery, yet of a sudden, by a perspiration breaking out, the eyes almost immediately have changed from a gloomy, heavy, or wild vacant stare, to a placid, yet lively, look; which, on such occasions, is the most conspicuous symptom of a favourable crisis having taken place. In such cases, the pulse, respiration, and other morbid symptoms, soon change accordingly, which is frequently followed by soft slumber. All this seems to denote that the constriction on the pores of the skin is suddenly removed, and also the obstruction to the circulation of the blood in the minute capillary vessels, both in the extremities and in the brain, and with this all appearance of putridity soon vanishes; which indicates that we are often too anxious to correct putrefaction, and give astringents and antiseptics, which in general increase, rather than remove, the obstructions before noticed, and which, *at all times*, should be our first and principal object of consideration. I admit that they may sometimes be usefully administered, particularly if joined with sudorifics and cordials; and if, by giving them, purgatives are not superseded.

It may be thought the height of presumption in me to find fault with the present nosology of fevers, formed, as it is, by very learned and scientific men, and, I believe, acknowledged by the generality of the profession to be as good as can be well adopted; yet I cannot help thinking it is capable of being made more simple and clear, rendering each class of fever more distinctly defined; which would be of essential service to all medical men, and particularly so to young practitioners. A theorist may fancy otherwise, and think they are already sufficiently clear; but in practice I think he will find it difficult to discriminate typhus mitior from typhus gravior, or synochus, (which, according to some authors, means the same;) and still more difficult to distinguish the former from synocha and common fevers, particularly in their latter stages; and which, in my humble opinion, is often the cause of bad practice.

Whenever we can distinguish any vital organ more affected than the rest, it appears to me reasonable and proper to make use of that organ as a distinguishing characteristic of such fever: as, for example, Phrensy, when the brain is particularly affected; Pleurisy, when the pleura is; and so on.

The term Bilious Fever is not in any nosology that I know of, yet it is often used in books and by medical gentlemen; but I do not think that it is sufficiently characteristic of any particular fever, for all fevers are more or less bilious: therefore, when the seat or cause of the fever appears to arise from the liver, would it not be better to add the name of the organ to fever, in such a manner as not to confound it with hepatitis, or local derangement? I merely throw out these hints to such of my brethren of the profession as are better qualified than myself to investigate the subject farther.

The following circumstances lately occurred, which I think will show clearly that typhous fevers are not always properly distinguished. A child, about a year and half old, of a full habit, and irritable disposition, was taken ill when cutting his two lower canine teeth: his pulse was quick, with a flushed face. The gums were lanced, and he took a laxative. The quickness of the pulse increased, and he had some slight convulsive spasms, and also frequent griping, with discharges of green watery stools, (when no purgatives were taken,) which were not particularly offensive. The quickness of his pulse continued rather to increase than diminish, and beat at the rate of 120 to 140 in a minute; yet his breath was always sweet, and skin soft, with no febrile heat, excepting one night when he took a small quantity of wine in gruel: in this state he continued till the ninth day, when I saw him, with two other medical gentlemen.

When he first saw us, his pulse beat from 160 to 170 in a minute, which increased action might in some measure be accounted for by the irritation produced on seeing strangers; for in a short time it sunk to 140. It is worthy of remark, that, at the time it beat quickest, there was little, if any, heat on the skin; the tongue and fauces were perfectly clean; and his breath was not the least offensive; nor were his evacuations more so than is usual with a child in health when taking mercurial purgatives. Notwithstanding all these favourable symptoms, he was thought to be labouring under typhus. Being myself of a different opinion, and considering his illness entirely owing to dentition, I did not see him after. He recovered in a few days, without much loss of substance.

It is proper to observe, that a child in the same house, a few years older, of the same disposition and full habit, was recovering from a fever when the little boy was first taken ill. The little girl I attended during the whole time of her illness; and, although she had some slight symptoms of typhus, such as fetid breath, fetid stools, and a little stupor, at times only, I do not consider that they were different from what is usual in common fevers, when they continue any length of time. One of the maid-servants was also ill at the same time with the little boy. I saw her, and her case appeared to be purely inflammatory, of which she recovered in a few days. Other servants were afterwards ill, but I cannot speak of their cases, as I did not see them: however, they soon recovered. A fever at the time prevailed in the town, chiefly amongst the lower orders of society, which alarmed the inhabitants exceedingly; and whether it was endemic or epidemic, I cannot say, as I had not many opportunities of seeing people in them. Two other cases I did see, which were perfectly distinct from any kind of typhus, yet by some they were considered as such. One of them was a clearly marked liver case; the other a chronic asthma, which, from great fatigue and watching, put on inflammatory symptoms, and was attended with considerable œdematous swellings of the legs. I believe almost all the disorders in the town at the time indiscriminately went by the appellation of typhus. I shall leave it to the consideration of the faculty, if this is not likely to produce improper treatment, and if the term is not somewhat ambiguous?

At the time the before-mentioned fever prevailed in the town, meat was exceedingly cheap. I attribute, in a great measure, the fever to that circumstance: poor people, who had not long been in the habit of eating much animal food, perhaps indulged

2

themselves to excess, the effect of which is obvious to every medical man. I believe that gentlemen's servants are often made ill by over-feeding, particularly when their services at night deprive them of their usual rest and sleep. Although several, in many families in the town, were ill at the same time, I could not learn that any person caught the fever by going to the houses of the sick; which makes me doubt its being infectious. The same cause which produced the fever in one of a family or town, might exist with all,—as any particular state of atmosphere, diet, &c. which might only affect such as were predisposed to receive its baneful influence. This, I fear, with medical men and others, is not always sufficiently considered. If a person has a fever, and another in the same house is soon after taken ill, it naturally occurs to our imagination that the second has received the infection from the first; as, until within late years, the generality of fevers were considered infectious. The majority of mankind even now consider common colds infectious, for the same reasons; and I account for it in like manner. One reason for fevers not being thought so infectious now as they were formerly, arises in a great measure from the attention paid to cleanliness, and the free admission of pure air.

Although, in all my practice, I have never witnessed any clearly marked cases of infectious fever, I am of opinion that such do sometimes occur: to me it seems as reasonable to conclude so, as to think that people are sometimes made ill by noxious vapours. It is a great misfortune that, in the theory of medicine, as in most other abstruse speculations, men of great talents and abilities are too often led away by their imagination in favour of some favourite scheme or theory, and soar beyond the bounds of probability. Because fevers are found not to be so generally infectious as was formerly supposed, some learned men of the profession think they are never infectious; which I conceive is going much too far. Although it is impossible to demonstrate otherwise, we have every reason to suppose that infectious miasma sometimes floats in the air, particularly the miasma of small-pox and measles; and, from analogy, it is reasonable to conclude that other disorders are communicated in the same manner, and our acting under a different impulse may be the cause of serious evils.

It will be remarked, that I have made use of a theory to account for the regular return of intermittent fevers, differing, I believe, from any before adopted; and the opinion I have given of continued fevers lasting to a certain state of putrefaction before a crisis takes place, is, I believe, also new, and differs considerably from the usual method of attributing it to the phases of the moon. I was led to these inferences by comparing fevers in this country with fevers in India, where I long resided. If their duration was governed by the moon, we might expect it alike in both countries, which is not the case: fevers in India are of a much shorter duration before a crisis takes place than in Europe, which I account for from putrefaction being more rapid: and it is worthy of remark, that, although it is more rapid, yet fevers there are very seldom infectious; therefore, I think putrefaction is not a satisfactory way of accounting for it, and some other should be sought for,—such as miasma.

During the time I resided at the Calcutta General Hospital, in a very unhealthy season, many bad fevers came into the hospital, principally from the ships at Diamond Harbour, and many of the men died soon after their admission. In a few instances, soldiers from the garrison died within a few hours from their first seizure, and numbers within twenty-four hours; yet there was not a single instance of the fever being communicated to the other patients in the hospital, or to any other person. I opened many of their bodies; but I can only give an account of their general appearance post-mortem from recollection. Their livers were all enlarged, some of them to an enormous size, and most of them had formed adhesions to the right side; the gall-bladders and ducts were gorged with dark-coloured bile. The spleens of all were diseased: in many it had more the appearance of grumous blood than a solid gland, which, when laid hold of, the fingers sunk into its substance. The intestines were almost full of a dark-coloured sordes, somewhat resembling chocolate grounds, which was extremely offensive. These were their most distinguishing appearances. I am sorry I cannot give a particular description of each case, having lost all memoranda on the subject.

*Great Torrington; 14th March, 1823.*

